# Coping with five mismatches between policy and practice in hemiboreal forest stands and landscapes

**DOI:** 10.1038/s41598-025-96836-4

**Published:** 2025-04-24

**Authors:** Michael Manton, Raimundas Petrokas, Šarūnas Kukcinavičius, Silvija Šaudytė-Manton, Charles Ruffner, Per Angelstam

**Affiliations:** 1 Vytautas Magnus University, Studentu Str. 13, Kauno r, Akademija, LT-53362 Lithuania; 2 Lithuanian Research Centre for Agriculture and Forestry, Institute of Forestry, Department of Forest Genetics and Tree Breeding, Kaunas, LT-53101 Lithuania; 3 School of Forestry and Horticulture, Southern Illinois University – Carbondale, Carbondale, IL 62901-4411 USA; 4 Department of Forestry and Wildlife Management, University of Inland Norway, Campus Evenstad, Koppang, N-2480 Norway

**Keywords:** Closer-to-nature forest management, Forest age, Forest disturbance regimes, Vegetation communities, Forest dynamics, Structural legacies, Sustainability, Boreal ecology, Conservation biology, Forest ecology, Forestry

## Abstract

Maintenance of forest ecosystems revolves around the long-term persistence and resilience of their components, structures and functions. Focusing on Europe’s hemiboreal forests, we evaluate mismatches between naturally dynamic forest ecosystems and current forest management systems forming obstacles for developing closer-to-nature forest management. Using Lithuania as a case study, we (i) quantify the main forest vegetation community types using soil types, ground layer flora, and tree and shrub species, (ii) review the relationships among these vegetation communities and their predicted natural disturbance regimes, (iii) analyse changes in tree species composition, (iv) compare the life expectancy of trees with harvest age, and (v) compare the contemporary stand age distributions with predicted natural disturbance regimes stand age distributions. Results show five mismatches between current practices and policy visions. Despite identifying 17 natural hemiboreal forest vegetation communities only eight dominant stand tree species were reported in current forestry reporting. The areal extents of three different natural disturbance regimes were: gap dynamics - mixed broadleaved forests on wet-mesic very fertile sites (22%), succession - mixed spruce forests on fertile sites (49%), and cohort dynamics - Scots pine forest on poor fertility sites (30%). Changes in tree species composition showed declines of primary tree species of 12–71% for the three disturbance regimes. The ratio of natural expected life expectancy to harvest age varied from two-fold to eight-fold across different tree species. Stand age distributions in naturally dynamic forests and managed forests revealed a current dramatic deficit of old-growth stands. Coping with the five identified mismatches between natural forests and current forest management requires multiple solutions: (1) closer-to-nature forest management that emulate natural disturbance regimes at tree and stand scales, (2) landscape planning, and (3) multi-level governance approaches.

## Introduction

For more than two centuries, European industrial forest management strategies have focussed on maximum sustained wood yield based on clear-felling that has resulted in even-aged stand rotations^1–3^. The focus on effective wood production has placed unprecedented pressure on biodiversity conservation^4^ and the delivery of other forest ecosystems services^5,6^. A wide range of studies have demonstrated that forests managed for wood production have led to radical alterations of forest ecosystems’ patterns and processes^7–13^. The reduction of structural legacies at multiple scales is one of the most distinguishing features of even-aged forests subject to clear-felling^14–16^. Additionally, this simplification and homogenisation of forest landscapes and stands has contributed to making Europe’s forests highly susceptible to disturbances (i.e., fire, drought, windthrow, pest outbreaks)^17–19^. Thus, not only the loss of ecological sustainability and forest biodiversity, but also the reduced resilience of forest ecosystems, has been highlighted.

Resilience is the capacity of an ecosystem’s ability to withstand and recover from disturbances while maintaining its natural patterns and processes^20,21^. When disturbances exceed an ecosystem’s resilience threshold, it may trigger a regime shift that leads to potentially irreversible changes in its composition, structure and functionality^22^. Thus, ecosystem resilience is a focal feature of sustainable resource management and conservation, because resilience can help ecosystems adapt to disturbances and climate change while sustaining biodiversity and ecosystem services^22^. Therefore, forest resilience is an additional focus of the European Union’s Green Deal to mitigate accelerating climate change and biodiversity loss^23^.

The EU Biodiversity Strategy for 2030 underscores the importance of enhancing forest resilience through sustainable management practices, reforestation with diverse and climate-adapted species^24^, and closer-to-nature forest management approaches^25,26^. Strengthening forest resilience not only supports ecological stability but can also reinforce socio-economic benefits for rural communities dependent on forest resources^27^. Thus, emulating natural dynamics and biodiversity conservation are foundations for coping with a wide range of forest vulnerabilities. For instance, forests composed of several tree species and structural elements, such as deadwood and old trees with microhabitats, are richer in biodiversity, are more resilient and more functionally diverse than those with only one tree species^28^.

The European Union’s recent Biodiversity Strategy^24^ and Forest Strategy^29^ represent iterated calls for sustainability including the development of closer-to-nature forest management^25^. Accordingly, the EU Forest strategy^29^ re-iterates previous European forest policy^30,31^, and posits sustainable forest management as the “*stewardship and use of forest lands in a way*,* and at a rate*,* that maintains their biodiversity*,* productivity*,* regeneration capacity*,* vitality and their potential to fulfil*,* now and in the future*,* relevant ecological*,* economic and social functions*,* at local*,* national and global levels*,* and that does not cause damage to other ecosystems*”. This stresses the need to sustain both natural patterns and processes of forest ecosystems, and that the conservation of native biodiversity is considered as an integral part of sustainable forest management.

The new visions toward securing multi-functional forests present many challenges and have stimulated hot debates about what sustainably managed forests and forestry are^32,33^. There are generally two conflicting views. First, the industrial forest sector manages their forests as a cropping system and produces high yields of raw materials for society’s use. Forest cropping systems aim to reduce the variability of different ecosystem components, such as age-classes within stands and forest management units, tree species diversity and sizes, as well as structural legacies and natural disturbances^34^. On the other hand, scientific, policy, and conservation communities advocate for an ecosystem-based approach, recognizing forests as complex, dynamic systems that provide a wide array of ecological, social, and economic benefits beyond timber production^1,35–37^. Striking a balance between these competing views is a central challenge in modern forest management, requiring landscape level integrative approaches that promote biodiversity, ecosystem resilience, and climate adaptation while ensuring forests continue to meet societal demands.

This study is about the implementation of policy relevant to forests. Policy reflect norms for what society wants^38^. The demand on forests has increased and now include new requirements, such as emulating forest naturalness (e.g., closer-to-nature forest management), spatial planning (e.g., triad landscape zoning), and stakeholders with diverse interests (e.g., social-ecological systems). The aim of this study is to explore mismatches between the norm of forest naturalness versus the outcomes of current forestry. Focusing on the hemiboreal forest ecoregion in Europe and using Lithuania as a case study we (i) quantify the main forest vegetation community types using soil types, ground layer flora, and tree and shrub species, (ii) review the relationships among these vegetation communities and their predicted natural disturbance regimes, (iii) analyse changes in tree species composition, (iv) compare the life expectancy of trees with harvest age, and (v) compare the contemporary stand age distributions with natural disturbance regimes stand age distributions. To support sustainability of Europe’s hemiboreal forests we discuss the need for novel closer-to-nature forest management, zoning by spatial landscape planning and the need for holistic learning through evaluation.

## Methods and materials

### Lithuania as a case study representing Europe’s hemiboreal forests

The hemiboreal forest ecoregion forms the transition zone between the boreal ecoregion in the north and the temperate ecoregion in the south, and can be found throughout Europe, North America and parts of Asia^39,40^. Globally, hemiboreal (or Acadian in North America)^41^ forests contain a mixture of boreal forest species, (e.g., *Pinus spp.*,* Picea spp.*,* Betula* spp., and *Populus spp.)*, and temperate broadleaved tree species (e.g., *Quercus spp.*,* Tilia spp.*, *Fraxinus spp.*, *Ulmus spp.*, *Carpinus spp. and Acer spp.*).

**Fig. 1:**
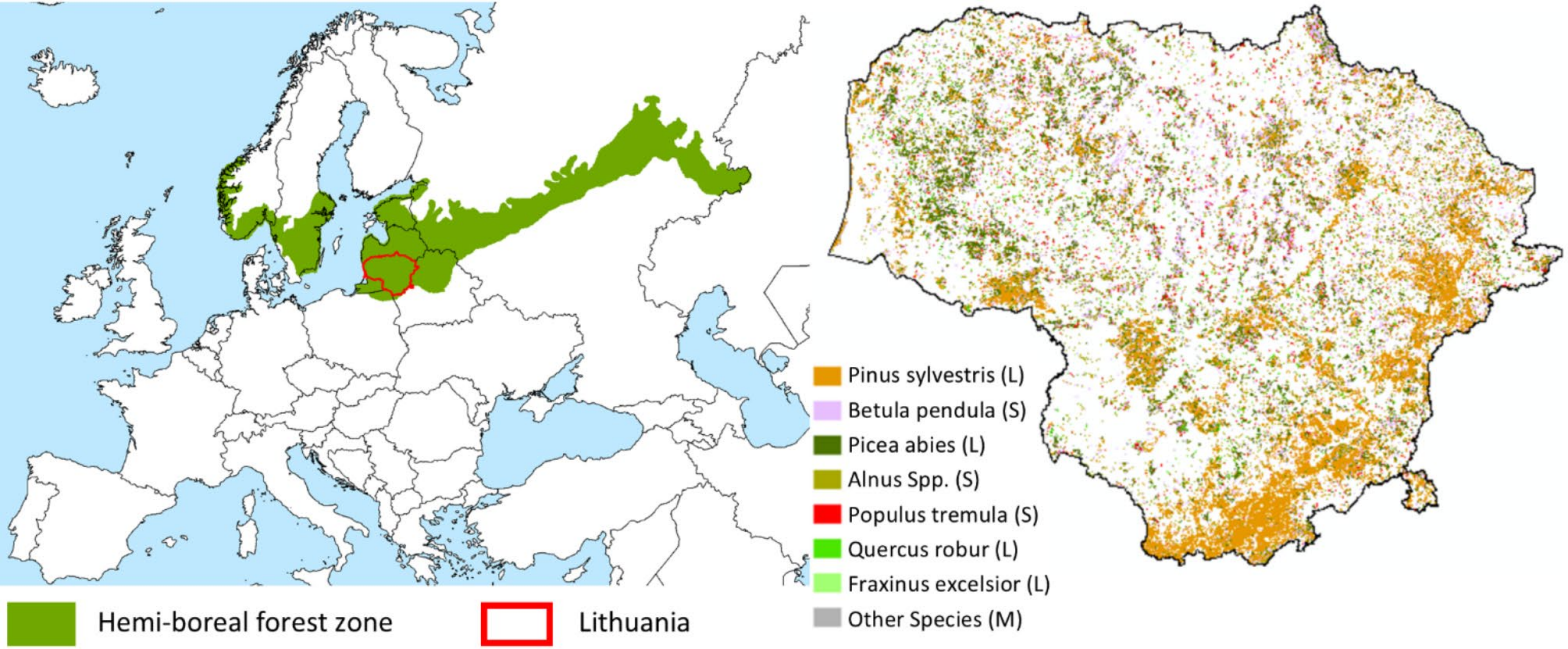
The distribution of the European hemiboreal forest zone^39^, and the location of Lithuania as the case study area of this review (left), and the distribution of dominant tree species in Lithuania^51^ (right). The letter following the tree species represents their life expectancy; S - short lived < 150 years, M – mixed ages, and L - long lived (see Fig. 3). The map was created using ArcGIS PRO 3.4.0 (https://www.esri.com/en-us/arcgis/products/arcgis-pro/overview).

Lithuania lies completely within the European hemiboreal forest ecoregion, has a wide range of representative forest site types, and was thus chosen as a case study^42^ (Fig. 1). Furthermore, forest site and stand data is available for analyses. Thus, Lithuania is ideal to explore the mismatches between current forest management regimes and new policy advocating emulation of natural forest disturbance regimes. According to the State Forest Service^43^, Lithuania’s total forest land area was 2,197,100 ha (34% of the total land area) in 2021. Scots pine (*Pinus sylvestris*) was the dominant species (35%) followed by silver birch (*Betula pendula*) (22%), Norway spruce (*Picea abies*) (21%), alder (*Alnus glutinosa* and *A. incana*) (14%), Eurasian aspen (*Populus tremula*) (5%), English oak (*Quercus robur*) (2%), ash (*Fraxinus excelsior*) (< 1%), and other species (1%) (Fig. 1).

### Exploring mismatches

#### Hemiboreal forest vegetation communities

Forest ecosystems are characterized by a range of different types of stand development, created by a range of forest-forming processes and disturbances that interact with the life-history characteristics of different tree species^44–47^. Tree species are adapted to the supply of water, nutrients and other environmental factors of the site and landscape, and have different means of dispersal, regeneration modes, competitive ability and patterns of recruitment in relation to different disturbances^48,49^. According to Olden^50^, species-specific approaches can predict species’ membership of a plant community, while still respecting different functional relationships among species. Maintaining multi-functional forests through long-term sustainable forest management highlights the importance of identifying representative forest vegetation communities and their complexity.

To understand the types and amounts of different hemiboreal forest vegetation communities in Lithuania, we analysed the Lithuania State Forest Service’s stand cadastral database^51^. Adhering to previous research on forest classification^47,52,53^, we first quantified the natural forest vegetation communities using five forest vegetation characteristics, viz.: forest site type, forest type series (field layer flora), dominant and secondary tree species, and shrubs. Second, we compared these with the current forest types presented in Lithuania’s national forest statistics^43^.

#### Hemiboreal forest disturbance regimes

Forest disturbance regimes are characterized by the type and magnitude of environmental variation following a disturbance event, as well as individual species adaptations and resistance to post disturbance conditions^46,54,55^. Forest disturbances vary in terms of spatial and temporal extents, frequency of occurrence and magnitude or intensity, and are influenced by the physical and biological conditions which affects the subsequent successional patterns of forest species^56,57^.

The use of natural disturbance regimes as benchmarks in forestry is grounded in their ability to maintain ecological processes, enhance forest resilience, and sustain biodiversity^36,58^. The disturbance regimes of the hemiboreal forest zone range from small-scale gap dynamics associated with small gaps in the canopy created by the loss of single trees, to stand-replacing disturbances, and cohort dynamics with partial lower intensity disturbances^14^. Forests can be classified into three main natural disturbance regimes based on climatic, topographic, edaphic and hydrological conditions^36,44^, namely gap dynamics, succession and cohort dynamics (Table 1). These three regimes provide reference conditions for incorporating natural forest dynamics into forest management at multiple spatial scales^7,14^.

**Table 1:** Summary of the characteristics of natural forest disturbance regimes in European hemiboreal forest.

Characteristics	Gap dynamics	Succession	Cohort dynamics	References
Site Conditions	Fertile soils Wet-Mesic	Moderate fertile soils Mesic	Poor infertile soils Dry sandy (dried out peatland)	^7,70^
Disturbance Extent	Small-scale disturbances	Large stand-scale disturbances	Partial disturbances	^14,56^
Disturbance Agent	Lightning, pathogens, treefall, ungulates	Insects, pathogens, fire, windthrow	Fires, insects, pathogens, storm damage	^19,108^
Disturbance Frequency	Low	High	Medium	^44,70^
Disturbance Severity	Low	High	Variable	^7^
ASIO fire frequency model	**A**bsent – 300 years **S**eldom – 100–150 years	**I**ntermediate – 80–100 years	**O**ften – 40–60 years	^7,19,70^
Dominant Tree Species*	Broadleaved deciduous	Early successional deciduous species followed by *Picea abies*	*Pinus sylvestris* with *Betula spp.* and *Quercus robur*	^47^
Forest Structure	Uneven-aged, high biodiversity, structural complexity	Even-aged, low structural diversity during early stages	Mixed-aged, mosaic with vertical and horizontal diversity	^14,108^
Regeneration Pattern	Regeneration under canopy; shade-tolerant species	Pioneer species dominate initially, later replaced by shade-tolerant species	Patchy regeneration, coexistence of old, mature, and young cohorts	^61,67^
Forest Management Strategy	Single stem and small group selection, nurse-tree shelterwood: selective harvesting of trees to create small gaps (< 0.5 ha); assisted natural regeneration based on maintaining a stabilized forest stand	Even aged stand replacement and retention forestry, irregular shelterwood: clear felling that retain both living trees and deadwood, and the replanting of mixed species; assisted natural regeneration	Multi-layer, tree retention, group shelterwood: partial harvesting of 10–60% of trees to create multi-aged stands with a continuous canopy cover, low intensity burning promoting regeneration; assisted natural regeneration based on competition	^7,26,36^

*See Table 3 for a full list of species.

To determine the spatial potential natural distribution of these three disturbance regimes in Lithuania, we identified and allocated each of the occurring plant communities, into one of the three disturbance regimes. First, we used the regional forest site series classification, based on soil moisture and fertility^52,59^, and the associated vegetation communities^52^, and hemiboreal tree species successional situation^47^to categorize Lithuania’s forest communities into the respective forest disturbance regimes. These were then mapped using the Lithuanian State Forest Service^51^ stand cadastral database in ArcGIS Pro.

#### Changes in species composition

Using the categorization of Lithuania’s natural forest communities and current forest vegetation distribution, we analysed the estimated changes in forest tree species composition. First, following Karazija^52^, we estimated the areas of primary tree species for each of potential natural vegetation communities and the three disturbance regimes. Second, we analysed the current areas of primary forest tree species, secondary forest tree species and other tree species for each of the vegetation communities using the Lithuanian State Forest Service^51^ stand cadastral database. Primary forest tree species are defined as the main stable forest composition developed under natural forest dynamics based on forest site types, secondary forest tree species are defined as the species that would form naturally under natural disturbances following major change to the primary forest tree species, and other tree species are those not defined as a primary or secondary forest tree species based on forest site type dynamics^52^.

#### Life expectancy of trees in natural and managed systems

As an indicator of maintaining the presence of tree species with different life history traits, we reviewed the natural life expectancy of Lithuania’s hemiboreal forest trees and compared them with the minimum and recommended harvest ages of production forests as defined by Lithuanian harvest regulations^47,60^. As a quantitative proxy of the proportional losses of old trees under contemporary forest management aiming for high yield wood production we divided the tree expectancy age by the harvest age for each tree species.

#### Stand development and age class distributions

Another important aspect to consider is stand development. According to Oliver and Larson^55^, there are four stages of stand development following stand-replacing disturbances, viz.: (i) young forest initiation - after a disturbance occurs, new individuals and species regenerate for several years; (ii) middle-age stem exclusion - when all growing space is occupied, no new stems become established and some trees die; (iii) old (mature) forest understory re-initiation - when new individuals emerge; (iv) old-growth stage - when overstory gaps created by dead and dying trees are filled with ingrowth. Table 2 illustrates the linkages between the three disturbance regimes (gap dynamics, succession, and cohort dynamics) and stand development phases. The analysis of disturbance regime and stand development showed that hemiboreal forest species can fulfil singular to multiple stand development positions (Table 2). For instance, birch and Eurasian aspen are shade intolerant pioneer species that primarily occur at stand initiation through all disturbance regimes. In contrast, intermediate shade intolerant species like Norway spruce, naturally occurs at the later stem exclusion and understory re-initiation stages of stand development within the three disturbance regimes.

**Table 2:** Forest stand development for the three disturbance regimes and the shade tolerance of Lithuania’s hemiboreal forest tree species following Oliver and Larson^55^.

Disturbance regimes	Stand development			
	Young forest initiation	Middle aged stem exclusion	Old (mature) forest understory re-initiation	Old-growth
Gap dynamics on wet-mesic very fertile sites	*Betula pendula*^1^ *(aeg cal cmh fil ur)* *Betula pubescens*^1^ *(aeg c cal cir cmh fil ur)* *Populus tremula*^1^ *(aeg cmh)* *Salix alba (ur)* *Salix fragilis*^1^ *(aeg ur)* *Alnus glutinosa*^2^ *(aeg c cal cir cmh fil ur)* *Alnus incana*^2^ *(aeg cmh fil)*	*Betula pendula*^1^ *(aeg cal cmh fil ur)* *Betula pubescens*^1^ *(aeg c cal cir cmh fil ur)* *Populus tremula*^1^ *(aeg cmh)* *Salix spp.*^1^ *(aeg ur)* *Quercus robur*^1^ *(aeg cal cmh)* *Alnus glutinosa*^2^ *(aeg c cal cir cmh fil ur)* *Alnus incana*^2^ *(aeg cmh fil)* *Fraxinus excelsior*^2^ *(aeg cmh fil ur)* *Picea abies*^2^ *(aeg c cal cir fil ur)* *Carpinus betulus*^3^ *(aeg)*	*Betula pendula*^1^ *(aeg cal cmh fil ur)* *Quercus robur*^1^ *(aeg cal cmh)* *Alnus glutinosa*^2^ *(aeg c cal cir cmh fil ur)* *Fraxinus excelsior*^2^ *(aeg cmh fil ur)* *Picea abies*^2^ *(aeg c cal cir fil ur)* *Tilia cordata*^2^ *(aeg cmh)* *Acer platanoides*^3^ *(aeg cmh)* *Carpinus betulus*^3^ *(aeg)* *Ulmus spp.*^3^ *(aeg cmh)*	*Quercus robur*^1^ *(aeg cal cmh)* *Fraxinus excelsior*^2^ *(aeg cmh fil ur)* *Picea abies*^2^ *(aeg c cal cir fil ur)* *Tilia cordata*^2^ *(aeg cmh)* *Acer platanoides*^3^ *(aeg cmh)* *Carpinus betulus*^3^ *(aeg)* *Ulmus spp.*^3^ *(aeg cmh)*
Succession on mesic fertile sites	*Betula pendula*^1^ *(hox mox ox oxn)* *Betula pubescens*^1^ *(oxn)* *Pinus sylvestris*^1^ *(hox mox ox)* *Populus tremula*^1^ *(hox mox ox oxn)* *Alnus glutinosa*^2^ *(oxn)* *Alnus incana*^2^ *(hox oxn)*	*Betula pendula*^1^ *(hox mox ox oxn)* *Betula pubescens*^1^ *(oxn)* *Pinus sylvestris*^1^ *(hox mox ox)* *Populus tremula*^1^ *(hox mox ox oxn)* *Quercus robur*^1^ *(hox mox ox oxn)* *Alnus glutinosa*^2^ *(oxn)* *Alnus incana*^2^ *(hox oxn)* *Fraxinus excelsior*^2^ *(oxn)* *Picea abies*^2^ *(hox mox ox oxn)* *Carpinus betulus*^3^ *(hox)*	*Betula pendula*^1^ *(hox mox ox oxn)* *Pinus sylvestris*^1^ *(hox mox ox)* *Quercus robur*^1^ *(hox mox ox oxn)* *Alnus glutinosa*^2^ *(oxn)* *Fraxinus excelsior*^2^ *(oxn)* *Picea abies*^2^ *(hox mox ox oxn)* *Tilia cordata*^2^ *(hox oxn)* *Ulmus spp.*^3^ *(hox oxn)* *Acer platanoides*^3^ *(hox ox oxn)* *Carpinus betulus*^3^ *(hox)* *Fagus sylvatica*^3^ *(hox)*	*Pinus sylvestris*^1^ *(hox mox ox)* *Quercus robur*^1^ *(hox mox ox oxn)* *Fraxinus excelsior*^2^ *(oxn)* *Picea abies*^2^ *(hox mox ox oxn)* *Tilia cordata*^2^ *(hox oxn)* *Acer platanoides*^3^ *(hox ox oxn)* *Carpinus betulus*^3^ *(hox)* *Fagus sylvatica*^3^ *(hox)* *Ulmus spp.*^3^ *(hox oxn)*
Cohort dynamics on dry infertile sites	*Betula pendula*^1^ *(m v vm)* *Betula pubescens*^1^ *(csp msp)* *Pinus sylvestris*^1^ *(cl csp lsp m msp v vm)* *Populus tremula*^1^ *(m vm)*	*Betula pendula*^1^ *(m v vm)* *Betula pubescens*^1^ *(csp msp)* *Pinus sylvestris*^1^ *(cl csp lsp m msp v vm)* *Populus tremula*^1^ *(m vm)* *Quercus robur*^1^ *(v vm)* *Picea abies*^2^ *(m vm)*	*Betula pendula*^1^ *(m v vm)* *Pinus sylvestris*^1^ *(cl csp lsp m msp v vm)* *Quercus robur*^1^ *(v vm)* *Picea abies*^2^ *(m vm)*	*Pinus sylvestris*^1^ *(cl csp lsp m msp v vm)* *Quercus robur*^1^ *(v vm)* *Picea abies*^2^ *(m vm)*

Superscript numbers indicates tree species’ regeneration shade requirements: ^1^ = shade intolerant, ^2^ = intermediate, and ^3^ = shade tolerant^47^. Forest type series (ground layer codes) are indicated in brackets(): *aeg*—*Aegopodiosa* (Nf), *c*—*Caricosa* (Pc), *cal*—*Calamagrostidosa* (Uc), *cir*—*Carico-iridosa* (Pd), *cl*—*Cladoniosa* (Nal), *cmh*—*Carico-mixtoherbosa* (Lf), *csp*—*Carico-sphagnosa* (Pb), *fil*—*Filipendulo-mixtoherbosa* (Ud), *hox*—*Hepatico-oxalidosa* (Nd), *lsp*—*Ledo-sphagnosa* (Pa), *m*—*Myrtillosa* (Lb), *mox*—*Myrtillo-oxalidosa* (Lc), *msp*—*Myrtillo-sphagnosa* (Ub), *ox*—*Oxalidosa* (Nc), *oxn*—*Oxalido-nemorosa* (Ld), *ur*—*Urticosa* (Uf), *v*—*Vacciniosa* (Na), *vm*—*Vaccinio-myrtillosa* (Nb).

We analysed and compared the current forest age class distributions^43^ against the predicted natural age class distributions for each of the three disturbance regimes (gap dynamics, succession, and cohort dynamics). The predicted natural distribution of age classes among disturbance regimes was derived from data and discussions presented by Berglund and Kuuluvainen^7^, Angelstam and Kuuluvainen^14^, Kuuluvainen and Gauthier^61^, and Pennanen and Kuuluvainen ^62^. The current forest age data were obtained from the State Forest Service stand cadastral database^51^. We used the following 40-year wide age class intervals 0–40, 41–80, 81–120, 121–160 and >161 years to describe the differences in stand age distribution profiles.

## Results

### Hemiboreal forest vegetation communities

The review of Lithuania’s current hemiboreal forest data identified 17 forest vegetation communities, and their relative proportions in the current forest cover. Of the 17 forest vegetation communities, four contributed to 67% of the total current forest cover in Lithuania. These were *Vaccinio-myrtillosa* – Scots pine forests (18%) and *Oxalidosa* – English Oak-Norway spruce forests (17%), *Myrtillo-oxalidosa* – Norway spruce forests (16%) and *Oxalido-nemorosa* – Norway spruce forests (16%). In comparison, the analysis of Lithuania’s national forest statistics showed forest management data for only eight dominant tree species at stand level, of which Scots pine (35%), Norway spruce (21%) and birch (22%) composed 78% Lithuania’s forests.

### Hemiboreal forest disturbance regimes

**Fig. 2:**
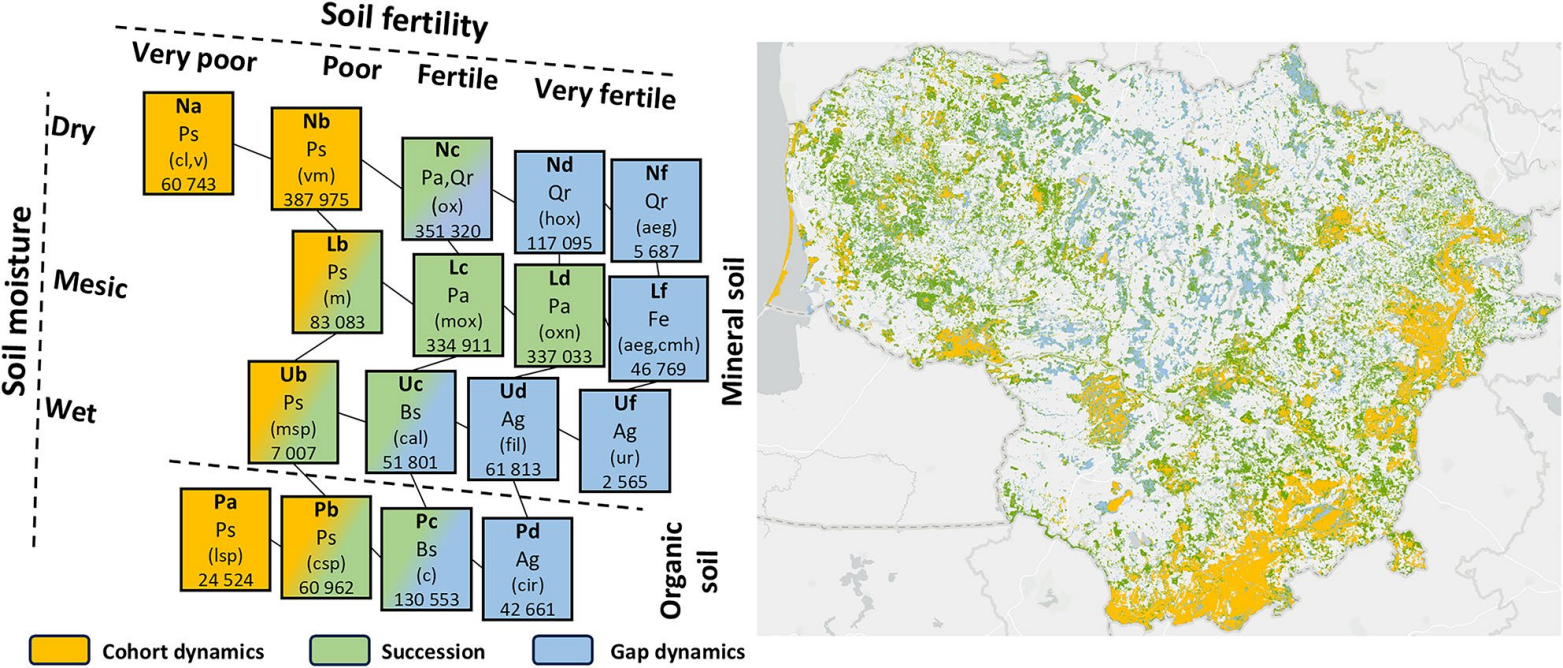
A snapshot of distribution of the three-disturbance regimes of Lithuania’s hemiboreal forests, viz. Gap dynamics with small, succession stand-replacing disturbance, and cohort dynamics with mixed age classes adapted from Petrokas et al., and Vaičys^53,59^. The bold codes refer to Lithuanian’s forest site types based on soil fertility and moisture, the small non-bold letters refer to the dominant tree species, and codes in the parenthesis denotes the ground layer flora^52^ and the number represent their area in hectares. The full range of Lithuania’s hemiboreal forest vegetation communities including soil type, ground layer flora, trees and shrubs can be found in Table 3. The dual colour boxes show that a given vegetation community can belong to two different disturbance regimes. Soil moisture: **N**—Dry soils, **L**—Mesic soils, **U**—Wet soils, **P**—Wet peatland, Soil fertility: **f**—very eutrophic soils, **d**—eutrophic soils, **c**—mesotrophic soils, **b**—oligotrophic soils, **a**—very oligotrophic soils. Dominant tree species: Ag – *Alnus glutinosa*, Bs – *Betula spp.*, Fe – *Fraxinus excelsior*, Pa – *Picea abies*, Ps – *Pinus sylvestris*, Qr – *Quercus robur*. Ground layer flora: aeg—*Aegopodiosa*, c—*Caricosa*, cal—*Calamagrostidosa*, cir—*Caricoiridosa*, cl—*Cladoniosa*, cmh—*Carico-mixtoherbosa*, csp—*Carico-sphagnosa*, fil—*Filipendulo-mixtoherbosa*, hox—*Hepatico-oxalidosa*, lsp—*Ledo-sphagnosa*, m—*Myrtillosa*, mox—*Myrtillo-oxalidosa*, msp—*Myrtillosphagnosa*, ox—*Oxalidosa*, oxn—*Oxalido-nemorosa*, ur—*Urticosa*, v—*Vacciniosa*, vm—*Vaccinio-myrtillosa*. The map was created using ArcGIS PRO 3.4.0 (https://www.esri.com/en-us/arcgis/products/arcgis-pro/overview).

Each of the types of forest vegetation community can be fitted to a soil fertility and soil moisture matrix, and to one or more of the three disturbance regimes (gap dynamics, succession and cohort dynamics) (Fig. 2). Gap dynamics contained eight vegetation communities on wet-mesic sites with high fertility, succession contained three vegetation communities on fertile soils, and cohort dynamics contained six vegetation communities on sites with poor soil fertility. The least common by area was gap dynamics (22%) with mixed broadleaved forests on rich sites, the most common was succession (49%) with even-aged spruce dominated forests on fertile site types. Cohort dynamics (30%) with Scots pine forest on dry infertile sites was in between (Fig. 2; Table 2). These three forest disturbance regimes were well distributed throughout Lithuania and matched the broad edaphic conditions (Fig. 2). Firstly, gap dynamics of mixed broadleaved forests were distributed in a central, north-south corridor of rich, alluvial soils left from the latest glaciation. Secondly, succession with mixed spruce dominated forests with fertile site types were identified in two areas, a large area of western Lithuania and a band running in a north-south direction in eastern Lithuania between the mixed broadleaved forests to the west and the Scots pine forest to the east. Finally, the cohort dynamics Scots pine forests were mainly located in eastern Lithuania on inland sand dunes or on various ombrotrophic wetlands as well as in multiple forest massive areas: Šimonys forest, Kazlų Rūda forest and Viešvilė forest.

**Table 3:** Main hemiboreal forest vegetation communities including soil type, ground layer flora, trees and shrubs of Lithuania. The forest vegetation communities are based on Karazija^52^ and classified using the Lithuanian state forest stand cadastral database^51^. For tree species, the **bold** text indicates the primary tree species, whereas normal font indicates secondary species that can also form mixed stands. See Fig. 2 for an overview of soil type categorisation and disturbance regime distribution.

Soil types (hydrotope-trophotope codes)	Ground layer flora	Tree species *	Shrubs **	Natural area, ha (%)	Current tree species, ha			Disturbance regime
					Primary	Secondary	Other	
Dry very fertile (Nd)	*Hepatico-oxalidosa (hox)*	***Qr *** *Pa Bs Pt Ai Cb Tc Fs*	*Sa Ca Lx Fa*	117095 (5.57)	21556	89036	6503	Gap dynamics
Dry very fertile (Nf)	*Aegopodiosa (aeg)*	***Qr *** *Fe Pt Ai Bs Cb Tc Ug Ul*	*Ca Sa Lx Fa Pa Ee Dm*	5687 (0.27)	660	4055	972	
Mesic very fertile (Lf)	*Aegopodiosa/Carico-mixtoherbosa (cmh)*	***Fe *** *Bs Qr Pt Ai Ag*	*Fa Ca Sa Pa*	46769 (2.23)	5402	32081	9286	
Wet fertile (Uc)	*Calamagrostidosa (cal)*	***Bs *** *Ag*	*Fa Sc Sa*	51801 (2.47)	22313	21051	8437	
Wet very fertile (Ud)	*Filipendulo-mixtoherbosa (fil)*	***Ag *** *Pa Fe Bs*	*Fa Sa Pa*	61813 (2.94)	39381	17588	4844	
Wet very fertile (Uf)	*Urticosa (ur)*	***Ag *** *Fe Bs*	*Pa Rn Fa Sa Ca*	2565 (0.12)	1711	621	233	
Wet fertile peatland (Pc)	*Caricosa (c)*	***Bs *** *Ag*	*Fa Sa Sc*	130553 (6.21)	71970	33212	25370	
Wet very fertile peatland (Pd)	*Carico-iridosa (cir)*	***Ag *** *Bs*	*Fa Sc*	42661 (2.03)	26267	13388	3007	
**Gap dynamics total**				**458944 (21.80)**	***189260***	***211032***	**58652**	
Dry fertile (Nc)	*Oxalidosa (ox)*	***Qr Pa *** *Ps Bs Pt *	*Sa Ca Fa Lx*	351320 (16.72)	64530	157130	129660	Succession
Mesic fertile (Lc)	*Myrtillo-oxalidosa (mox)*	***Pa *** *Bs Pt Ps Or*	*Sa Fa*	334911 (15.94)	153239	156328	25344	
Mesic very fertile (Ld)	*Oxalido-nemorosa (oxn)*	***Pa *** *Bs Pt Fe Qr Ai Ag*	*Sa Ca Fa Dm Lx*	337033 (16.04)	80860	251432	4741	
**Succession total**				**1023264 (48.70)**	***298629***	***564890***	**159745**	
Dry very poor (Na)	*Cladoniosa (cl) Vacciniosa (v)*	***Ps *** *Bs*	*Jc Sa*	60743 (2.89)	58617	1	2125	Cohort dynamics
Dry poor (Nb)	*Vaccinio-myrtillosa (vm)*	***Ps *** *Pa Bs Pt*	*Sa Fa Jc*	387975 (18.47)	366625	20993	357	
Mesic poor (Lb)	*Myrtillosa (m)*	***Ps *** *Pa Bs Pt*	*Fa Sa*	83083 (3.95)	51957	30973	153	
Wet poor (Ub)	*Myrtillo-sphagnosa (msp)*	***Ps *** *Bs Pa*	*Fa*	7007 (0.33)	4270	2733	4	
Wet poor peatland (Pb)	*Carico-sphagnosa (csp)*	***Ps *** *Bs*	*Fa Sc*	24524 (1.17)	23423	0	1101	
Wet very poor peatland (Pa)	*Ledo-sphagnosa (lsp)*	***Ps***	***-***	60962 (2.90)	42425	15183	3354	
**Cohort dynamics total**				**624295 (29.72)**	**547317**	**69883**	**7095**	

* *Ag* – *Alnus glutinosa* L. Gaertn., *Ai* – *Alnus incana* L. Moench, *Bs* – *Betula spp.*,* Cb* – *Carpinus betulus* L., *Fs* – *Fagus sylvatica* L., *Fe* – *Fraxinus excelsior* L., *Pa* – *Picea abies* L. Karst, *Ps* – *Pinus sylvestris* L., *Pt* – *Populus tremula* L., *Qr* – *Quercus robur* L., *Tc* – *Tilia cordata* Mill., *Ug* – *Ulmus glabra* Huds., *Ul* – *Ulmus laevis* Pall.

** *Ca – Corylus avellana* L., *Dm – Daphne mezereum* L., *Ee – Euonymus europaea* L., *Fa – Frangula alnus* Mill., *Jc – Juniperus communis* L., *Lx – Lonicera xylosteum* L., *Pa – Padus avium* Mill., *Rn – Ribes nigrum* L., *Sa – Sorbus aucuparia* L., *Sc – Salix cinerea* L.

### Change in species composition

**Fig. 3:**
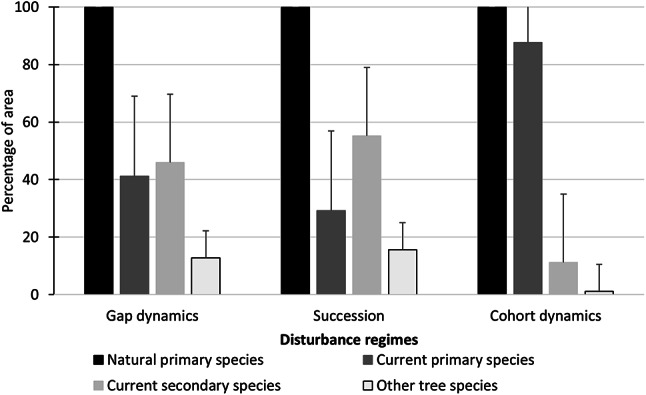
Changes in tree species composition from natural primary tree species to current primary forest tree species, second forest tree species, and other forest tree species.

The comparison of potential natural primary tree species to current primary, secondary, and other tree species varied across the three disturbance regimes. Under gap dynamics, there was a substantial shift away from natural primary species, with only 41% of the composition being retained as the current primary species. A large proportion (46%) was replaced by current secondary species, while 13% consisted of other tree species. The succession disturbance regime showed a similar trend, current primary species accounted for 29%, secondary species comprised of 55%, while other tree species was 16%. In contrast, cohort dynamics showed the highest retention of primary tree species (88%), with only 11% replaced by secondary species and 1% by other tree species (Fig. 3). This suggests that cohort dynamics have maintained a tree species composition similar to the original natural primary species, allowing minimal replacement by secondary or other species. Overall, the findings indicate that gap dynamics and succession have transitioned away from their natural primary species composition, with secondary species playing a dominant role, whereas cohort dynamics have largely sustained their primary species with minimal species turnover.

### Life expectancy of trees in natural and managed systems

**Fig. 4:**
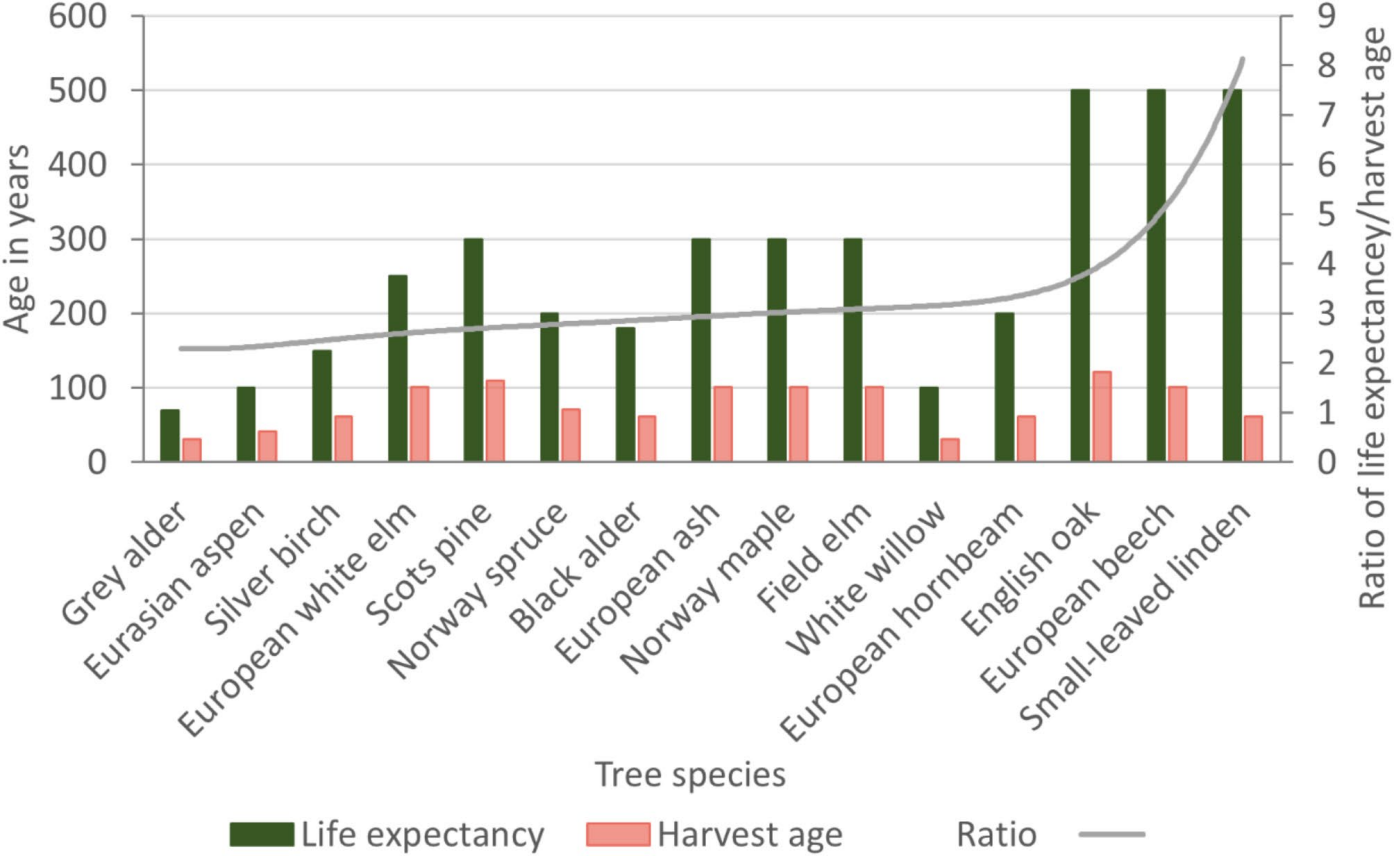
The natural life expectancy of Lithuania’s hemiboreal tree species compared to their nominated minimum harvest age for wood and the ratio of life expectancy divided by harvest age.

The analysis of mean natural life expectancy compared to the nominated harvest age in Lithuania reveals that final harvesting for all tree species occurs well before they reach their life expectancy and potential to provide key structural legacies supporting biodiversity and influence long-term successional dynamics (Fig. 4). For instance, both small-leaved linden and English oak can live up to ca. 500–600 years, but the harvest age is set at 80 and 110 years of age, respectively. For the dominant wood industry species of both Scots pine and Norway spruce the harvest age is set at 101 and 71, whereas their natural life expectancy is ca. 400 and ca. 300 years^47^, respectively.

The ratio of natural life expectancy to harvest age varies significantly across different tree species, reflecting disparities in management practices and ecological considerations. Fast-growing species such as grey alder and Eurasian aspen exhibited lower ratios of 2.3 and 2.4, respectively, indicating shorter life expectancies and earlier harvesting (Fig. 4). Most of the species, including the main timber species of Scots pine, Norway spruce and birch, showed ratios of 2.5–3.3. In contrast, long-lived species like English oak, European beech (*Fagus sylvatica*) and small-leaved linden showed substantially higher ratios of 4.1, 5.0 and 8.2, respectively.

### Stand development and age class distributions

**Fig. 5:**
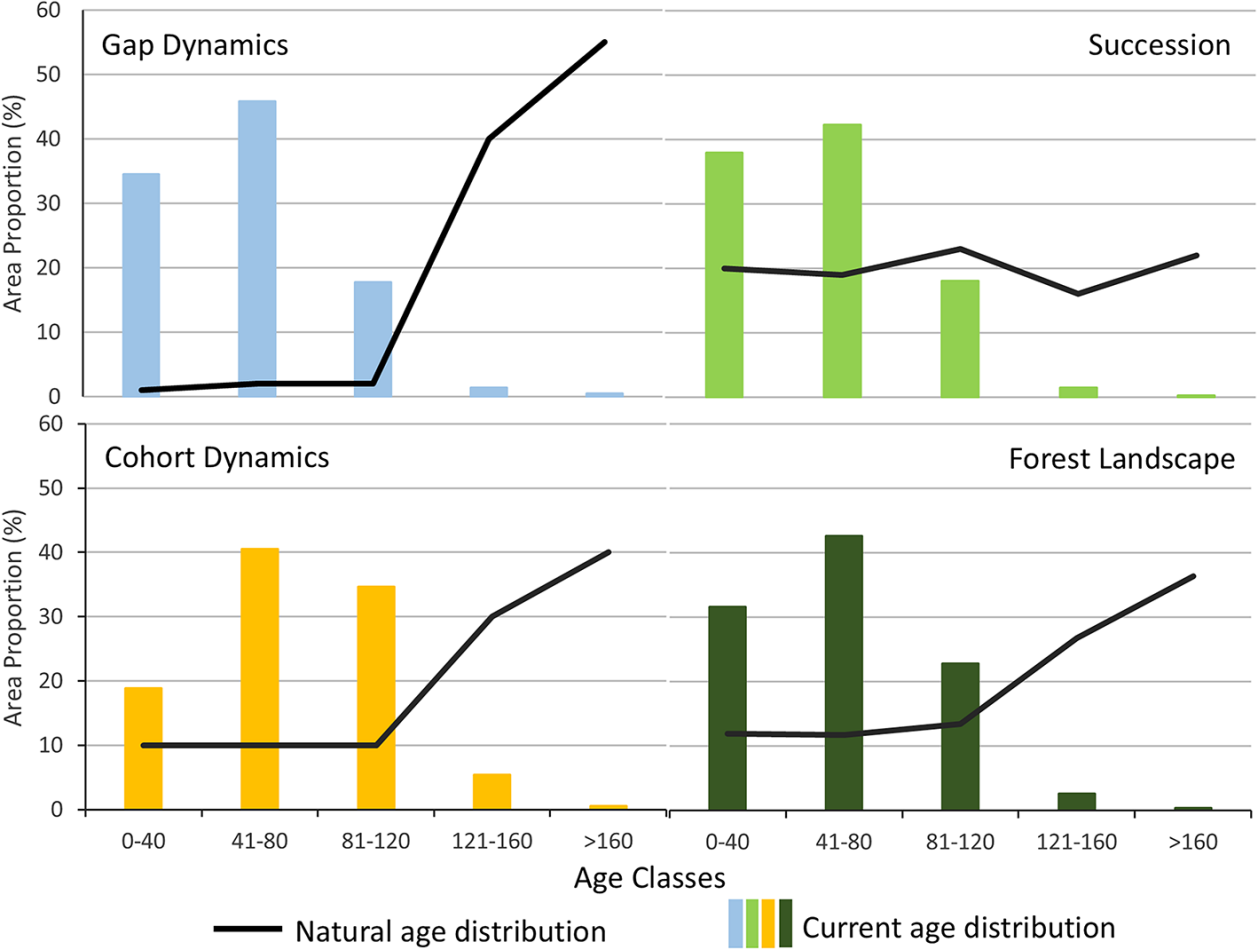
Forest stand age class distribution in Lithuania 2021 (vertical bars), and the expected forest disturbance regimes (lines) under the three disturbance regimes, and the total landscape stand age distribution. The natural age distribution estimates are based on data and deliberations^7,14,61,62^.

The results of the analysis of current age structure versus the three disturbance regimes exhibited large differences (Fig. 5). Firstly, the current range of forest stand age was limited to the three youngest classes < 120 years. This shows that even-aged rotation forestry using clear-felling systems is applied throughout Lithuania on all forest site types and across all three different potential forest type disturbance regimes.

Second, under a predicted natural age distribution for both gap dynamics and cohort dynamics there is a dominance of forest stands > 120 years, while for succession the age class distribution is relatively equal among the five 40-year wide age classes. We conclude that ageing and old forests > 120 years are severely underrepresented, particularly for gap and cohort dynamics. Combining these age class distributions with the amount of forest site class types suitable for different disturbance regimes shows the clear mismatch between current and natural age distributions in Lithuania (Fig. 5, bottom right).

## Discussion

### Mismatches between managed and naturally dynamic forest landscapes

In relation to current policies regarding sustainable forest management, biodiversity conservation and ecological resilience, our analyses demonstrate that significant mismatches persist in five key dimensions: hemiboreal forest vegetation community types, forest disturbance regimes, tree species composition, life expectancy of tree species and stand age distributions.

First, the vegetation community types analysis revealed that the hemiboreal forests of Lithuania host 17 different natural types. In contrast, the State Forest Service^43^ only reports on the eight dominant tree species stands in Lithuania, of which Scots pine (35%), Norway spruce (21%) and birch (22%) make up 78% of the forested area. To cope with this mismatch requires a transition from single dominant tree species stand reporting to the inclusion of the natural vegetation forest stand communities.

Second, the disturbance regime analysis showed the natural hemiboreal forests encompass three natural disturbance regimes: gap dynamics (22%), succession (49%), and cohort dynamics (30%) (Fig. 2; Table 3). The succession regime can be viewed as the principal inspiration and rationale of even-aged rotation forestry using clearcutting in Lithuania. This regime also includes thinning of midstory trees waiting to be recruited into mature tree canopies or overstories. In contrast, the broad diversity of vegetation communities with characteristic disturbance regimes suggests that forest management should employ multiple forest management strategies that vary in temporal and spatial scales and harvesting intensities, thus emulating each of the three disturbance regimes. For example, implementing a range of partial harvesting methods in cohort dynamic forests can foster structural diversity by providing a much-needed diversity of deadwood in various states of decay at a stand scale, and a mosaic of age and size classes at both stand and landscape levels. Partial harvesting in cohort dynamics forests includes the harvesting of 10–60% of the trees using multiple harvest methods, such as, selective cutting, shelterwood cutting, strip cutting, and thinning that create a multi-aged and structured forest landscape mosaic^46,63,64^. Whereas selective harvesting under gap dynamics is the cutting of single-trees, small group selections, or gap cuts with a maximum size of 0.5 ha that mimic natural disturbance patterns, as opposed to the clear-cutting of larger, contiguous areas^25^. Undertaking heterogenous selective and partial harvesting approaches in both gap and cohort dynamic forests would thus enhance forest stand connectivity, structural elements, forest age profiles and species habitats^65,66^ and deliver a wider range of ecosystem services other than wood^14^.

Third, the analysis on species composition showed large differences in primary tree species between natural forest and current managed forests for the forest regimes of gap dynamics (59% decline) and succession (71% decline). In comparison, the primary forest tree species under cohort dynamics have remained relatively close to natural condition with only a 12% decline. This indicates that current forest management has transformed the primary tree species of both wet-mesic very fertile sites and mesic fertile sites to secondary species and other species. The differences in primary species loss can be largely explained by current forest management practices prioritizing faster tree growth and high wood yields. Given that the potential natural vegetation in Lithuania is forest, approximately 64% has been lost to agriculture and urbanization^67^.

Fourth, the analysis on tree age showed the expected natural longevity of Lithuania’s hemiboreal tree species was, on average, two-fold to eight-fold higher compared to the current minimum harvest age. The ratios were particularly high for English oak, beech and small-leaved linden. This pattern highlights mismatches between the natural longevity of these tree species and current forest management regulations. Current minimum tree harvest ages are defined by species and forest management groups of which commercial group IV forests consist of 72% of the total forest area. This indicates that the relationships between site types, tree growth and tree age could be further developed under closer-to-nature forest management. In addition, the increased demands for new wood products and processing methods have significantly increased the use of lower ages and wood grades globally^68^. This intensified use underscores the need to modify harvest practices by allowing sufficient amounts or trees to develop old-growth characteristics and microhabitats^69^. Prioritizing the conservation and restoration of old-growth forest elements across the landscape is thus urgently needed.

**Fig. 6:**
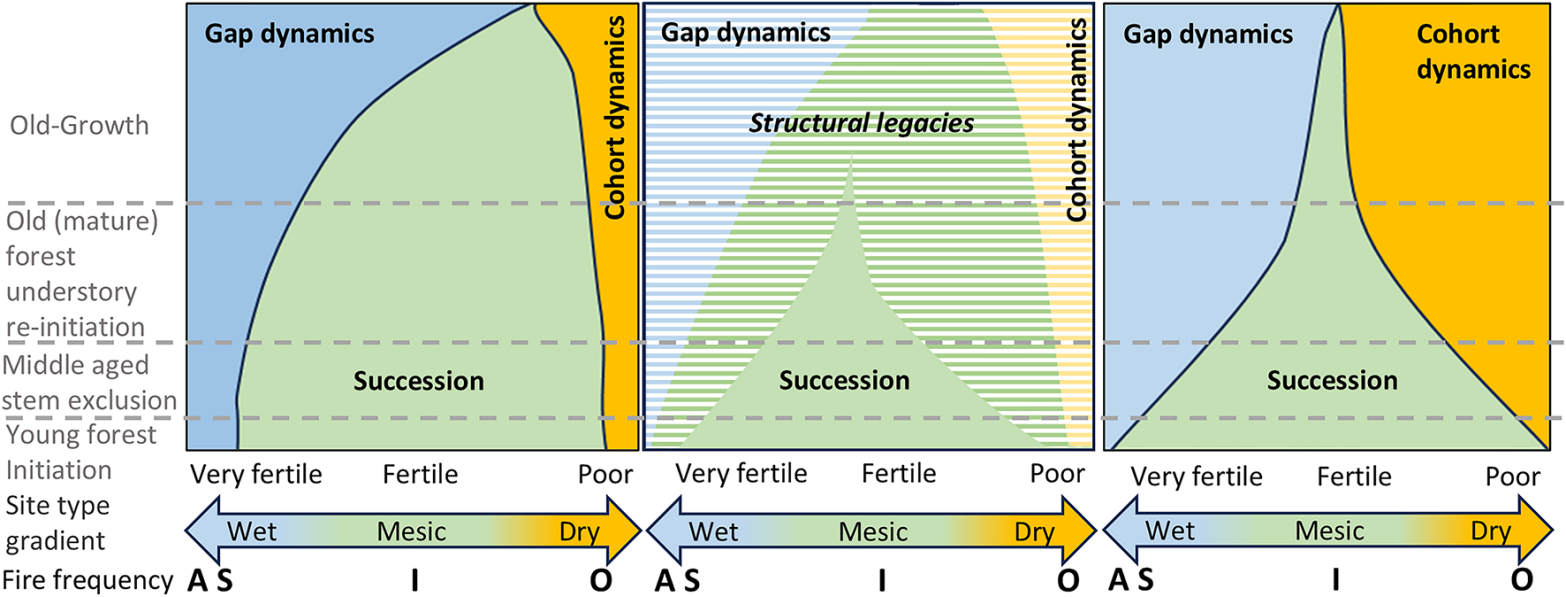
Two perspectives on the relative proportions of natural disturbance regimes in relation to the linkages between the range of forest site types from wet to dry on the one hand and stand development stages linked to time since disturbance on the other. The hatched areas represent structural legacies from previous disturbance events. One perspective is based on time since disturbance and implicitly also the accumulation of structural legacies^70^ (left), and the other focuses on only time since disturbance without accumulated structural legacies^7^ (right). The illustration in the middle captures both perspectives. Thus, both these two perspectives highlight the occurrence high amounts of old-growth forests across disturbance regimes in the natural system. The fire frequency refers to the ASIO fire frequency model: Absent, Seldom, Intermediate and Often^70^.

Fifth, the analysis on stand age distributions in naturally dynamic and managed forests showed that there is a significant shortfall of old-growth forests (Fig. 5). The so called ASIO model, based on the relative fire frequences **A**bsent, **S**eldom, **I**ntermediate and **O**ften, visualises the linkages between the range of forest site types from wet to dry on the one hand, and stand development stages on the other^70^ (Fig. 6 left). While earlier development stages are dominated by successional dynamics in an average landscape, the proportion of the landscape occupied by the old-growth stage over time should become dominant across all disturbance regimes. This is linked to increasing time since disturbance and is combined with increasing amounts of structural legacies maintained over multiple disturbance events. Focusing on only time since disturbance, without considering accumulated structural legacies, Berglund and Kuuluvainen^7^ proposed an alternative model (Fig. 6 right), and argued that the ASIO-model leads to an underestimation of the amount of later seral stages with old-growth characteristics. Adding accumulation of structures at successive disturbance events increases the total amount of old-growth stand characteristics. The middle illustration in Fig. 6 captures both perspectives. Thus, both models highlight the occurrence of high amounts of old-growth forests in the natural system. Consequently, the conclusion that there is a deficit in the amount of old forest across the landscape is clear and robust. This is valid even if the estimates of natural age distributions are tentative. For example, the expected amount of natural age classes > 120 are several times higher than the current forest age structure (Fig. 5). Thus, even imprecise estimations do not impact the conclusion. We stress the need to adjust harvest ages to represent the natural life expectancy of trees.

Overall, given current policy documents relevant for forests, our analysis of the five mismatches reveals an over-simplification of Lithuania’s current forest ecosystems. This is driven by a focus on industrial wood production using clearcutting rotation forestry. The Lithuanian State Forest Service^71^ reported that in 2020, 94% of final harvests were clearcuts, while the remaining 6% were classic seed-tree harvests. This leaves seed trees at a stand stocking level of 0.2–0.3 ha^−1^. Seed trees are then removed when regeneration has been secured. This results in even-aged forest stands with no remaining natural legacies or older stand structures. Neither, continuous cover forestry nor shelterwood systems are applied^72^. Thus, in Lithuania the focus of traditional forest management is based on the ‘’Normalwaldmodel” (normal forestry model) which is overused and has been critiqued in Western Europe^2,34,73^. The normal forest model attempts to rationalise forests and make them predictable, quantifiable and visible as a resource and thus facilitates the management and extraction of even-aged clear-felling rotations to maximise sustained yield and the economic benefits^74^. This has favoured a limited number of commercially valuable species, mainly Scots pine, Norway spruce and birch, and thus short forest rotations. The above-mentioned forestry practice is aggravated by increasing harvest rates of Lithuania’s forests, which have doubled from 3.0 to 6.7 mil. m^3^of wood between 1990 to 2020^43,75^. Moreover, this has led to an estimated mean age of 53 years^2^, to a 3-fold reduction of species reliant on structural legacies^76^ and losses in the availability of mature old-growth forest for large nesting birds^77^ in Lithuania. Nonetheless, Pihlajamäki 2018, even called for an additional 15% increase in yearly harvesting by clear-felling without any environmental consideration^78^. Thus, later successional stages are essentially not found nor their associated lichen, fungi, and coarse woody debris dynamics typical of old stage successional seres^79^. This illustrates the fundamental difference between natural forests and policy visions recognizing the diverse characteristics of forest ecosystems on the one hand, and the current practices of the forest industry that is focused on the economic benefits on the other hand^78,80^. This is also the situation in other European countries with hemiboreal forests^6,81^. To cope with these five mismatches there is a need for new approaches to forest management, spatial forest planning and learning though evaluation.

### Closer-to-nature forest management

The tasks of effective sustained yield wood production focussing of industrial raw material typical for Europe’s hemiboreal forest, and maintenance of natural ecological patterns and processes of naturally dynamic forest ecosystems, are not compatible^6,81^. Over recent decades, policy about sustainable forest management, conservation of forest ecosystems and biodiversity, as well as multi-functional forest landscapes have stimulated the development of a wide range of forest management terms, forest management practices and visions for future sustainable multi-functional forest management^82^. For instance, the new concept of “closer-to-nature forest management”^25,29^ is inspired by natural disturbance regimes, and is misleadingly associated to the term “close to nature forestry”^9,83^. Although they are similar in name there is an inherent difference in their aims and level of forest modification required to emulate different levels of forest naturalness. Instead, close to nature forestry is rooted in continuous cover forestry practices for central European gap dynamic forests^83^. It seeks to lightly balance ecological and economic priorities with the later given priority. In contrast, closer-to-nature forest management is driven by modern conservation knowledge^36^ and new EU policy^48^.

**Fig. 7:**
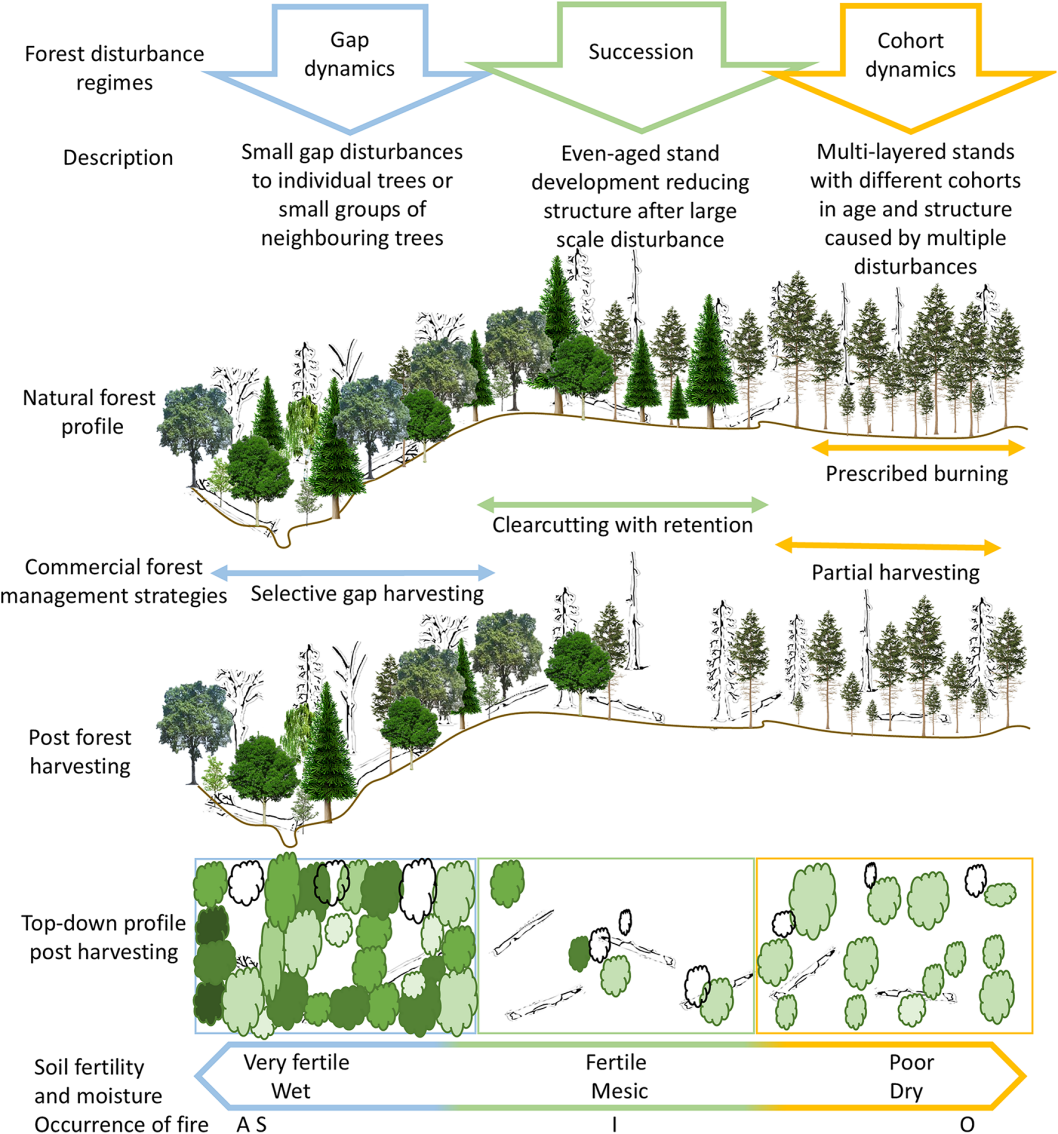
Conceptual drawing of successional pathways towards closer-to-nature forest management and emulation of the three natural forest disturbance regimes for hemiboreal forests based on soil fertility and moisture. The top shows the natural forest disturbance regimes: gap dynamics (blue), succession (green) and cohort dynamics (orange)^7,14,36^ and how forest management can implement cutting strategies (middle) that emulate natural disturbance regimes and their outputs on the ground (bottom). The different coloured green trees on the top-down profile post harvesting represent different species and sizes. The occurrence of fire refers to the ASIO fire frequency model: **A**bsent, **S**eldom, **I**ntermediate and **O**ften^70^.

In conclusion, our results support the EU Forest Strategy^29^ calling for the diversification of forest management systems to deliver multi-functional forest landscapes through emulation of the three natural forest disturbance regimes for hemiboreal forests (Fig. 7). As a guideline for Europe’s hemiboreal forests, this should be based on using natural characteristics of site fertility and moisture, forest tree and ground vegetation communities, forest disturbance regimes as well as tree and stand age structure profiles. The life history trajectory of deciduous broadleaved hardwood trees in Europe’s hemiboreal forest should naturally regenerate on wet-mesic very fertile soils under gap dynamics using selective harvesting. Only stands with a disturbance regime of succession on mesic fertile soils with a primary species of Norway spruce should be subject to even-aged rotation forestry. Cohort dynamic on poor fertile soils with Scots pine forest stands require partial, or intermediate harvesting that maintain a multi-aged structure, as well as vertical and horizontal diversity and complexity. In addition to coping with the five mismatches, sufficient retention of structural legacies, such as dead wood, microhabitats and structures linked to old trees, are needed in all disturbance regimes^69,84^.

### Zoning by landscape planning

In addition to closer-to-nature forest management, securing functionally connected habitat networks, and effective wood production, require different approaches to forest landscape planning^85^. Landscape zoning, such as the triad approach, addresses mismatches between policy and forestry practices by designing conservation, timber production, and mixed-use zones^86–88^. This strategy minimizes trade-offs, aligning ecological and socio-economic priorities for sustainable resource management. Savilaakso, et al.^89^ showed that forests > 80 years old are important to maintain biodiversity in boreal forests and that natural forests are needed to ensure the conservation of forest core dependent species. Importantly, both protected and voluntary set-aside areas of natural forest remnants are needed to ensure conservation of species dependent on old-growth and primary forests^80,89,90^.

A key question is how much should be allocated to each of these zones? Blattert, et al.^91^ explored a range of scenarios of the three triad zones and evaluated how different relative proportions impacted landscape multi-functionality defined by biodiversity and ecosystem service indicators. Their results show that maximizing multi-functionality required around 20% wood production, 50% mixed use, and 30% conservation. Adjusted from the past Soviet forest management system, Lithuania forest management employs a four-zone planning approach^92,93^. This approach is similar to the triad forest zoning approach^87^ as it provides *strict nature protection* (Group I - < 2%), two groups that share the management objectives of both wood protection and nature protection; *special purpose forests* (Group II – 12%) and *protective forests* (Group III − 15%), and finally *commercial forests *(Group IV − 72%) for wood production^43^. To safeguard European forests the EUs Biodiversity Strategy^24^ has placed more emphasis on the protection of forest ecosystems by nominating a 10% strict protection and 20% voluntary protection targets by 2030, leaving 70% for wood production. Thus, international conservation policy mirrors the evidence-based “third-of-third” rule of thumb approach with a third of the land area managed as multi-use conservation (33%) within which a third (11%) is strictly protected and the rest (67%) is used for production^94^.

However qualitative aspects also need to be satisfied (e.g., habitat quality and functional connectivity)^95^. Thus, conservation may need strict protection, conservation management and nature restoration which combine both passive and active actions. This is illustrated by the low proportion of strict nature protection (< 2%) in Lithuania, thus highlighting the current shortfall of old-growth forests. This situation is similar in neighbouring Latvia, Poland and Sweden^85,96^. To increase the quality and quantity of old-growth forest for protection will require landscape planning, conservation management and nature restoration, which are supported by European Union’s closer-to-nature forest management, as well as the recent Nature Restoration Law^97^.

Landscape strategic and tactical planning tools, such as gap analysis^98,99^ and habitat suitability index modelling^96^, respectively, can be used to guide decision-making. Gap analysis assesses forest representativeness and deficiencies in existing protected area networks by evaluating whether habitats and species are adequately represented and safeguarded^100^. Habitat suitable index models use spatially explicit land cover data and quantitative requirements of focal species to predict and map key areas with optimal environmental conditions and forest habitat connectivity^65,101^.

### Learning to cope with mismatches

Coping with the five mismatches between natural forests as a benchmark for closer-to-nature forest management, and current forest management practices identified in this study requires viewing forests as social-ecological multi-level systems. This involves diverse spatial and temporal scales^102^, as well as multiple levels of forest and landscape governance^103^. At the tree and stand scale, closer-to-nature forest management emerges as a practical solution, emphasizing management practices that emulate natural disturbance regimes, promoting biodiversity conservation, and the maintenance of ecosystem services. At the landscape scale, zoning by landscape planning offers a promising solution to addressing mismatches between ecological patterns and processes, and forest management practices^85^. The triad zoning strategy allows for creating a balance between different aspects of sustainable forest management at the landscape level^86,91^. A major unresolved challenge, however, is to plan across ownership and administrative borders. Effective forest management and landscape planning in Europe requires a multi-level governance approach that integrates the social system at different levels, from international policies via national regulations to local practices^104^. Collaborative governance, involving representative stakeholders, can bridge the gap between policies and practical implementation, enhancing adaptive and inclusive forest management^105^. Forest management practices are based on knowledge, norms and social relations acquired in specific geographical contexts over a long time^1,106^. This may lead to inertia and barriers for learning new ways of management and planning in forest landscapes. This stresses the need for integrating learning in science and practice among policy makers and the public. Accounting for all the benefits and disservices of ecosystem services is also needed to provide comprehensive comparison of outcomes resulting from changes in forest management that can be measured. However, to maintain multi-functional forest landscapes through the development and establishment of management models that respond to the needs of industry, climate and biodiversity crises is not easy. Learning through evidence-based evaluation about how conditions and trends develop is one way forward, but tangible outcomes of these are often missing^107^.

## Data Availability

The datasets used and/or analysed during the current study is available from the corresponding author on reasonable request.
